# Giant thymoma presenting as a large bilateral intrathoracic mass: A case report and a comparison between median sternotomy and hemiclamshell approach

**DOI:** 10.1016/j.amsu.2021.102859

**Published:** 2021-09-13

**Authors:** Daoud Daoud, Bassam Darwish, Sarmad Zahra, Monir Qaddoura

**Affiliations:** a Faculty of Medicine, Damascus University, Damascus, Syrian Arab Republic; b Department of Thoracic Surgery, Al-moassat Hospital, Damascus, Syrian Arab Republic

**Keywords:** Giant thymoma, Bilateral thoracic mass, Hemiclamshell, Median sternotomy

## Abstract

**Introduction:**

Thymoma is an epithelial tumor that commonly lies in the anterior mediastinum. It rarely extends to the pleural cavities. There is no standard approach for resecting similar giant thymomas.

**Case presentation:**

An eighteen-year-old woman presented with a six-month history of progressive exertional dyspnea, weight loss, and loss of appetite. Radiological imaging demonstrated a giant mediastinal mass extending to both pleural cavities, a transthoracic needle biopsy was then performed, which indicated thymic hyperplasia.

**Clinical discussion:**

The tumor was completely resected using a two-step approach, starting with a median sternotomy then extending it to a hemiclamshell incision, which provided better exposure of the tumor and caused less morbidity.

The left part of the thymoma was resected using a median sternotomy, which took a relatively long time and caused significant blood loss. Then the incision was extended to a hemiclamshell incision through the pleural cavity to remove the right part of the tumor. This approach helped us to visualize the tumor better and did not cause any significant blood loss.

The removed mass measured 36 × 29 × 10 cm and weighed 4500 g. Pathologic diagnosis indicated a type B1 tumor with no capsular invasion according to the World Health Organization classification.

**Conclusion:**

The hemiclamshell approach is superior to the median sternotomy incision in resecting giant thymomas extending to the pleural cavity, as it saves time and causes less morbidity.

## Highlights

• Giant Thymoma is an extremely rare clinical entity.
• It is very uncommon for a thymoma to reach both pleural cavities.
• Comparing the median sternotomy to the hemiclamshell approach in resecting giant thymomas extending to the mediastinum.
• The hemiclamshell approach caused less morbidity and no significant blood loss compared to the median sternotomy.

## 1 Introduction

Thymoma mostly originates in the anterior mediastinum. Giant intrathoracic thymomas are infrequently reported, and none in the medical literature was reported to extend to both pleural cavities. Surgery is the treatment of choice, and no adjuvant therapy is usually required. Although median sternotomy is the operative approach of choice to excise normal-sized thymomas, there is no consensus in the medical literature on the best surgical incision for giant thymomas.

In this paper, we report a case of a giant intrathoracic thymoma that was resected through a median sternotomy and hemiclamshell approach, through which we provide a comparison between these two different incisions.

This work has been reported in line with the SCARE criteria [21].

## 2 Case presentation

An eighteen-year-old female presented to the clinic with a six-month history of progressive exertional dyspnea, weight loss, and loss of appetite. The patient's BMI was 15. She had no relevant personal or family history, no reported allergies, and never smoked. Chest examination revealed dullness to percussion, markedly absent breath sounds to auscultation and absence of respiratory vibrations to palpation over most parts of the left and right hemithoraces. The patient had no clinical signs of myasthenia gravis.

**Fig. 1:**
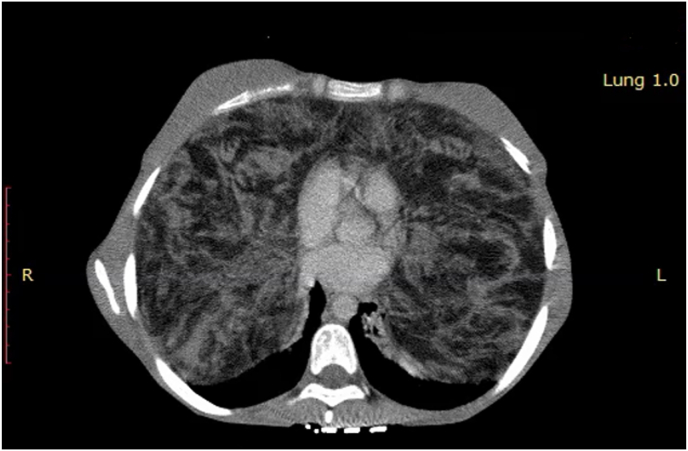
Patient's chest CT: a mass taking up most parts of both chest cavities and measuring 36 × 29 × 10 cm.

Chest CT demonstrated a giant bilateral non-cystic mass with a heterogeneous enhancement in the thoracic cavities that presses posteriorly and superiorly against the lungs. It had no calcification (Fig. 1). Laboratory studies were unremarkable. A transthoracic needle biopsy was performed, which revealed thymic hyperplasia. Thymoma was suspected, and surgical resection was recommended.

A median sternotomy incision was our first choice as surgical access in order to isolate the mass from the innominate vein. The left part of the tumor was resected from outside the pleura. Bleeding occurred during this approach, and three units of blood were transfused. Due to the difficulties and the excessive blood loss with the first excision through a median sternotomy, we used the hemiclamshell approach to resect the right part of the tumor, in which we extended the median sternotomy through the 5^th^ intercostal space to the right pleural cavity in order to make an additional anterolateral incision. This approach provided a very good exposure, through which a total resection of the right part was performed. Using this approach, we managed to perform the operation in less time, and it was easier to detect the right phrenic nerve and the right lung hilum while causing less bleeding than with the median sternotomy approach alone.

**Fig. 2:**
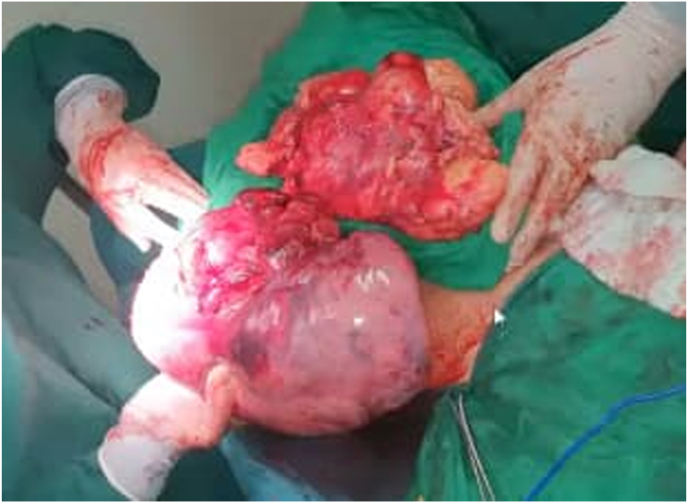
Gross specimen of the mass.

**Fig. 3:**
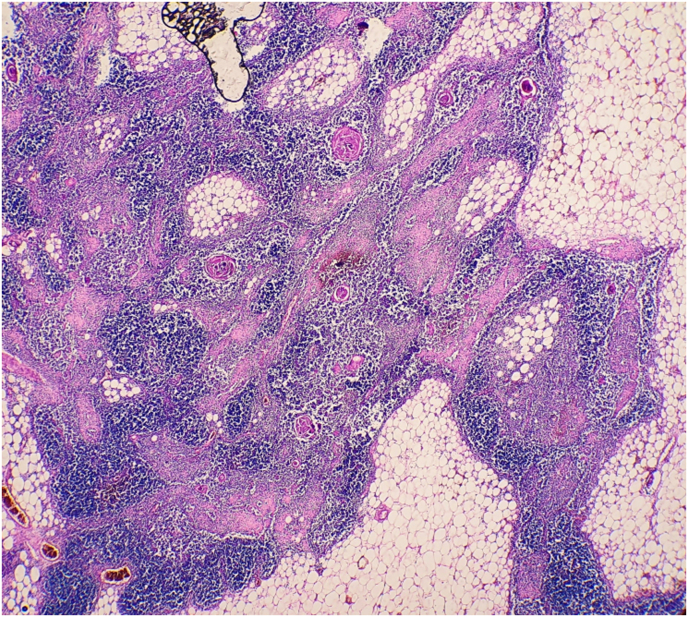
Histopathological findings from the postoperative specimens (x 40).

**Fig. 4:**
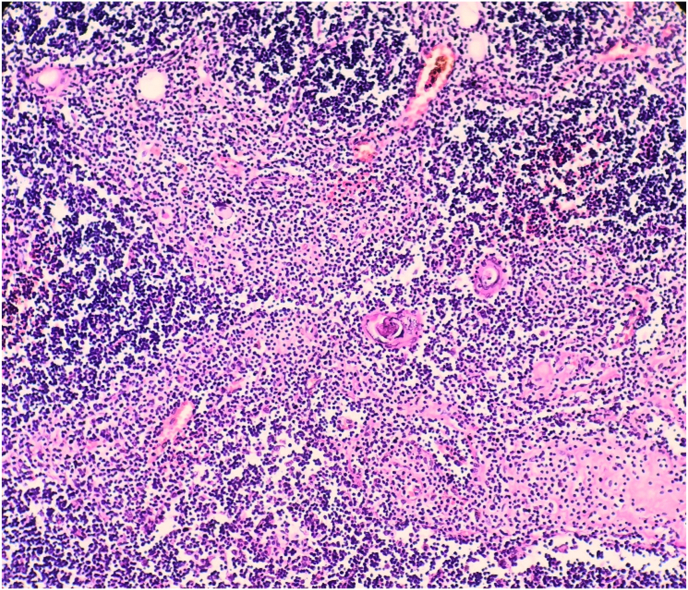
Histopathological findings (x 100) showing diffuse proliferation of thymic epithelial cuboidal cells with Hassall corpuscles and a large amount of mature mainly T-lymphocytes.

The resected specimen was 36 × 29 × 10 cm in size and weighed about 4500 g (Fig. 2), and showed well-circumscribed contours and slightly lobulated yellow cross-sections with scattered foci of ischemia. Microscopic examination revealed diffuse proliferation of thymic epithelial cuboidal cells with Hassall corpuscles and a large amount of mature mainly T-lymphocytes (Fig. 3, Fig. 4). The diagnosis indicated by the histopathological findings as lymphocyte-predominant thymoma (Type B1 according to the World Health Organization classification). The patient got weaned off after three days from mechanical ventilation, and the postoperative course was good.

## 3 Discussion

Thymoma is a slow-growing epithelial neoplasm, accounting for about 20–30% of all mediastinal tumors [1]. Thymic tumors represent 0.2–1.5% of all malignancies with an incidence of 0.15 per 100,000 of the population and equally affect males and females [2]. Giant intrathoracic tumors are infrequently reported in the literature, and none was reported to extend bilaterally to both pleural cavities.

Thymomas are usually asymptomatic, but they may have local symptoms such as chest pain, cough, and dyspnea due to the compression of the surrounding organs. Superior vena cava syndrome and weight loss occur with more aggressive tumors [3]. Some patients may have another systemic autoimmune disease, such as myasthenia gravis or, less commonly, hypogammaglobulinemia and pure red cell aplasia [4]. In this case, the patient had dyspnea and severe weight loss.

The differential diagnoses of a giant intrathoracic mass include sarcoma, solitary fibrous tumor, lymphoma, mesothelioma, and metastatic tumors. A Transthoracic core needle biopsy accurately differentiates thymic tumors from other intrathoracic masses and has high sensitivity and specificity (90–100%) [5]. Although the transthoracic biopsy indicated thymic hyperplasia, not thymoma, both are indications of surgical resection.

The median sternotomy is the operative approach of choice to excise normal-sized thymomas [6], but there is no consensus in the medical literature on the best approach to remove giant thymomas that extend to the pleural cavities. Only a median sternotomy was used in a few cases [7,8], as the innominate vein was invaded. The anterolateral thoracotomy was the operation of choice in some cases [[9], [10], [11], [12]], allowing the extension of the incision to include a posterolateral or a hemiclamshell approach. Posterolateral thoracotomy was also used in three cases [[13], [14], [15]], but this approach has bad access to the anterior mediastinum. Because of that, it was followed in Ref. [14] by a separate median sternotomy to resect the thymus gland completely. The Hemiclamshell approach is also a practical approach for giant thymomas. It provides better exposure of the upper thoracic cavity and the pulmonary hilum, and it can be a second choice approach by extending a median sternotomy with an anterolateral thoracotomy or vice versa. The Hemiclamshell approach was used in several cases [1,[16], [17], [18], [19]], including the present case. Only one case used the clamshell incision in an emergency operation [20], which is a more invasive approach.

In this case, we resected the left part of the tumor using the median sternotomy approach without opening the pleural cavity, which took more time and was complicated by a relatively massive blood loss. Whereas extending the incision to a hemiclamshell approach led to an easier resection of the right part of the tumor without any significant bleeding, and we could easily detect the phrenic nerve and the pulmonary hilum on the right side.

## 4 Conclusion

It is extremely rare for a thymoma to reach the pleural cavity on both sides, so it is mandatory to exclude a wide range of differential diagnoses before making the definitive diagnosis. We also recommend extending the usual median sternotomy to a hemiclamshell approach by making an anterolateral incision overlying the 5^th^ intercostal space and entering through the pleura, as it saves time, reduces bleeding, and provides better exposure of the phrenic nerve and pulmonary hilum, thus providing a safer intervention for the patient.

## Ethical approval

No ethical approval was needed.

## Sources of funding

There was no funding.

## Author contribution

DD: reviewed the literature, wrote the case presentation, and the introduction. BD; led the surgical team, revised the manuscript, and helped in writing the discussion. MQ: reviewed the literature, checked for spelling and grammar, and helped in writing the discussion. SZ: reviewed the literature, wrote the abstract, and the discussion.

## Registration of research studies

N/A.

## Guarantor

Mr. Sarmad Zahra.

## Consent

Written informed consent was obtained from the patient for publication of this case report and accompanying images. A copy of the written consent is available for review by the Editor-in-Chief of this journal on request.

## Provenance and peer review

Not commissioned, externally peer-reviewed.

## Declaration of competing interest

All of the authors declared that they have no conflict of interest.

## References

[1] Zhao W., Fang W. (2016). Giant thymoma successfully resected via hemiclamshell thoracotomy: a case report. E677-80J. Thorac Dis.

[2] Engels E.A., Pfeiffer R.M. (2003 Jul 1). Malignant thymoma in the United States: demographic patterns in incidence and associations with subsequent malignancies. Int. J. Canc..

[3] Kim H.J., Cho S.Y., Cho W.H. (2013). An unusual case of superior vena cava syndrome caused by the intravascular invasion of an invasive thymoma. Tuberc. Respir. Dis..

[4] Briones J., Iruretagoyena M., Galindo H. (2013). Thymoma associated with hypogammaglobulinaemia and pure red cell aplasia. Ecancermedicalscience.

[5] Bilaçeroğlu S. (2020). How to obtain adequate biopsy specimen in suspected thymic tumors. J. Thorac. Dis..

[6] Tomaszek S., Wigle D.A., Keshavjee S. (2009). Thymomas: review of current clinical practice. Ann. Thorac. Surg..

[7] Fazlıoğullari O., Atalan N., Gürer O., Akgün S., Arsan S. (2012). Cardiac tamponade from a giant thymoma: case report. J. Thorac. Cardiovasc. Surg..

[8] Spartalis E.D., Karatzas T., Konofaos P., Karagkiouzis G., Kouraklis G., Tomos P. (2012). Unique presentation of a giant mediastinal tumor as kyphosis: a case report. J. Med. Case Rep..

[9] Limmer S., Merz H., Kujath P. (2010). Giant thymoma in the anterior-inferior mediastinum. Interact. Cardiovasc. Thorac. Surg..

[10] Yamazaki K., Yoshino I., Oba T., Yohena T., Kameyama T., Tagawa T. (2007). Ectopic pleural thymoma presenting as a giant mass in the thoracic cavity. Ann. Thorac. Surg..

[11] Saito T., Makino T., Hata Y., Koezuka S., Otsuka H., Isobe K. (2015). Giant thymoma successfully resected via anterolateral thoracotomy: a case report. J. Cardiothorac. Surg..

[12] Alexiev B.A., Yeldandi A.V. (2016). Ectopic pleural thymoma in a 49-year-old woman: a case report. Pathol. Res. Pract..

[13] Filosso P.L., Delsedime L., Cristofori R.C., Sandri A. (2012). Ectopic pleural thymoma mimicking a giant solitary fibrous tumour of the pleura. Interact. Cardiovasc. Thorac. Surg..

[14] Gotte J.M., Bilfinger T.V. (2007). Resection of giant right-sided thymoma using a lateral thoracotomy approach followed by median sternotomy for completion thymectomy. Thorac. Cardiovasc. Surg..

[15] Aydin Y., Sipal S., Celik M., Araz O., Ulas A.B., Alper F. (2012). A rare thymoma type presenting as a giant intrathoracic tumor: lipofibroadenoma. Eurasian J Med.

[16] Azuma Y., Otsuka H., Makino T. (2018). Giant thymoma successfully resected via median sternotomy and anterolateral thoracotomy: a case report. J. Cardiothorac. Surg..

[17] Takenaka T., Ishida T., Handa Y., Tsutsui S., Matsuda H. (2012). Ectopic thymoma presenting as a giant intrathoracic mass: a case report. J. Cardiothorac. Surg..

[18] Tsubota N., Murotani A., Yoshimura M. (1993). A huge non-invasive thymoma causing acute dyspnea. Tohoku J. Exp. Med..

[19] Takanami I., Takeuchi K., Naruke M. (1999). Noninvasive large thymoma with a natural history of twenty-one years. J. Thorac. Cardiovasc. Surg..

[20] Santoprete S., Ragusa M., Urbani M., Puma F. (2007). Shock induced by spontaneous rupture of a giant thymoma. Ann. Thorac. Surg..

[21] Agha R.A., Franchi T., Sohrabi C., Mathew G., Kerwan A. (2020). The SCARE 2020 guideline: updating consensus surgical CAse REport (SCARE) guidelines. Int. J. Surg..

